# EFFECTIVENESS OF TELEDELIVERED BEHAVIORAL ACTIVATION AND MINDFULNESS INTERVENTIONS IN REDUCING LONELINESS

**DOI:** 10.1093/geroni/igad104.2722

**Published:** 2023-12-21

**Authors:** Da Jiang, Dannii Yeung, Namkee Choi, Rainbow Tin Hung Ho, Jojo Yan Yan Kwok, Youqiang Song, Lisa M Warner, Kee Lee Chou

**Affiliations:** Education University of Hong Kong, Hong Kong, Hong Kong; City University of Hong Kong, Kowloon Tong, Hong Kong; University of Texas, Austin, Texas, United States; The University of Hong Kong, Hong Kong, Hong Kong; The University of Hong Kong, Hong Kong, Hong Kong; The University of Hong Kong, Hong Kong, Hong Kong; MSB Medical School Berlin, Berlin, Germany; Education University of Hong Kong, Hong Kong, Hong Kong

## Abstract

Loneliness is one of the most prevalent mental health problems in older adults, especially among those who live alone and are digitally excluded. The phenomenon has become more conspicuous during the COVID-19 pandemic because of the constraints on physical contact and travel. In this three-armed randomized controlled trial, we examined the effectiveness of two telephone-delivered loneliness interventions (i.e., behavioral activation [Tele-BA], mindfulness [Tele-MF]) against a telephone-delivered active control group (i.e., befriending [Tele-BF]) in reducing loneliness, based on the behavioral theory of depression and monitor acceptance theory. We trained retirees as lay counselors to deliver the interventions to enhance the scalability and sustainability of the interventions. Older adults who lived alone and under the poverty line in Hong Kong, did not have Internet at home, and felt lonely were randomly assigned to one of the three groups (N = 1045, Mage = 76.59, SDage = 7.83, 78% female). Participants in each group received two 30-minute weekly intervention sessions for four weeks. They completed assessments at the baseline (T1), four weeks (T2) and six months (T3) following the intervention, respectively. Compared with their counterparts in the Tele-BF group, participants in the Tele-BA and Tele-MF groups reported lower levels of loneliness and perceived stress and higher levels of psychological well-being and perceived social support at T2 and T3. These findings provide scientific understandings on the effectiveness of Tele-BA and Tele-MF interventions for reducing loneliness among older adults in Hong Kong, and demonstrate the feasibility of conducting lay counselor-delivered telephone-based interventions for older adults.

